# Decoding intervention for young students with mild intellectual disabilities: A single-subject design study

**DOI:** 10.1177/17446295231208819

**Published:** 2023-11-11

**Authors:** Linda Fälth, Heidi Selenius, Christina Sand, Idor Svensson

**Affiliations:** Department of Pedagogy and Learning, 4180 Linnaeus University, Sweden; Department of Special Education, 7675 Stockholm University, Sweden; Department of Psychology, 4180 Linnaeus University, Sweden; Department of Psychology, 4180 Linnaeus University, Sweden

**Keywords:** student, decoding, intellectual disabilities, intervention, single-subject design

## Abstract

Students with intellectual disabilities need more time and explicit instruction to develop word decoding. Most previous research on interventions among these students is performed in English. Therefore, the current study examined the impact of a word-decoding intervention in Swedish on individual students with intellectual disabilities. A single-subject-design study was conducted with five students with mild intellectual disability in the fourth grade. They needed to enhance decoding, and Swedish was their first language. Their word and non-word decoding was measured during the baseline and intervention phases. The intervention with the Wolff Intensive Program was delivered by special education teachers supporting phonemic decoding and reading fluency training during 25 sessions. All five students developed their decoding as they decoded more words in a given time (NAP=0.84-1.00) and decreased their decoding errors in both word and nonword decoding (NAP=0.72-1.00). The results are promising but need to be confirmed in additional studies.

## Introduction

Being able to read is a prerequisite for independent coping in society. For students with intellectual disabilities, learning to read is often challenging, and reading performance is often lower than expected based on their individual cognitive level (Channell et al., 2013; Conners, 2003; Ratz & Lenhard, 2013). Compared to students with other disabilities and peers without disabilities, students with intellectual disabilities usually have less well-developed reading (Afacan et al., 2021; Channell et al., 2013; Wei et al., 2011). The technical part of reading, i.e., decoding, may be difficult to acquire for students with intellectual disabilities due to cognitive and learning challenges. These students may need additional support and explicit instruction in decoding to improve their reading ability (Afacan et al., 2021).

Generally, reading research shows that systematic decoding training is required to acquire a functional reading ability (Snowling & Hulme, 2012). Students’ skill to decode or read single words strongly determines their overall reading ability (Gough & Tunmer, 1986; Stanovich, 1991). Consequently, monitoring and supporting the students’ decoding might improve their reading ability. A useful model in reading education for understanding the developmental stages and strategies students use to learn to decode is put forward by Frith (1985). Her model comprises three stages of decoding development: logographic, alphabetic, and autonomous. In the logographic stage, students are visually oriented and rely on rote memory to recognize words. They use any visual cues available to them, such as the overall shape of the word or the position of the letters within the word, to associate the written word with its meaning. This stage is characterized by a reliance on whole-word recognition rather than phonemic analysis. In the next stage, the alphabetic stage, students will begin to understand that words can be broken down into smaller units called phonemes, representing the individual sounds that make up a word. The students learn that phonemes (letter sounds) are represented by graphemes (letters), and they begin to use phonemic analysis to decode words. Finally, in the autonomous stage, students become proficient in phonemic analysis and can use this skill automatically and efficiently to decode words. With this skill, the students have the prerequisites to read with fluency and comprehension, using context and other sources of information to understand the meaning of new words. This stage is characterized by automaticity in reading and a more sophisticated understanding of language and literacy. Students with intellectual disabilities may have a delayed reading development, so they might need support progressing beyond the alphabetic stage (Frith, 1985; Lundberg et al., 1988).

In reading education using phonics, the students are taught to identify letters and their sound correspondences and to sound them out to decode words (Ehri, 2022). Meta-analyses on intervention performed among students without intellectual disabilities and who struggle with decoding have reported positive effects of individualized phonics instructions (Al Otaiba et al., 2023). Similarly, research has shown that interventions focused on improving decoding skills, such as phonemic awareness training, can be beneficial in supporting students with intellectual disabilities to learn to read more proficiently (Katims, 2001). Studies have also shown that students with intellectual disabilities can enhance decoding skills as a result of intensive and systematic phonics instruction in English (Allor et al., 2014; Fredrick et al., 2013; Tucker et al., 2008). Explicit instruction in phonemic awareness and phonics is also demonstrated to be useful in improving reading fluency and comprehension among students with intellectual disabilities (Allor et al., 2014). Compared to students in mainstream schools, many students with intellectual disabilities require more explicit instructions and a longer time when learning to decode words (Afacan et al., 2018; Alquraini & Rao, 2020; Canella-Malone et al., 2015). Also, a more significant teacher presence is required for students with intellectual disabilities in reading education (Alquraini & Rao, 2020; Canella-Malone et al., 2015).

Still, as the phonemic and phonological structures of different languages can differ significantly (Fuchs et al., 2013), these promising results from decoding interventions in English among students with intellectual disabilities might be inappropriate to generalize to different languages and education systems. For example, compared to English, the Swedish language has several vowel sounds, consonant sounds, and diphthongs (Fuchs et al., 2013). Consequently, students’ development of decoding skills in English and Swedish might differ. Besides, in the Swedish educational system, students with intellectual disabilities attend compulsory education for ten years (Swedish National Agency for Education, 2011), which differs from the education system in the US (Cavanagh, 2017; National Center for Education Statistics, 2020). Students with intellectual disabilities in Sweden follow a different curriculum than their peers without an intellectual disability. The *Curriculum for compulsory school for pupils with intellectual disabilities* differs from the *Curriculum for compulsory school*, as it is tailored to meet the needs of students with various disabilities or learning difficulties. For example, when it comes to the subject of Swedish, these formulations regarding strategies are added in the *Curriculum for compulsory school for pupils with intellectual disabilities*: for understanding and making oneself understood include techniques such as paraphrasing, using images, and employing gestures or signs; to reproduce parts of the content in different texts and reason about the message and action in texts. Also, compared to the *Curriculum for compulsory school*, assessment and grading criteria differ and are developed to meet the various disabilities and learning difficulties among students with intellectual disabilities (Swedish National Agency for Education, 2011).

Subsequently, as languages and educational systems differ (Fuchs et al., 2013), there is a need for research on interventions focusing on word decoding skills in Swedish to provide students with intellectual disabilities with efficacious reading education. Therefore, the current study aimed to examine the impact of a word-decoding intervention in Swedish on individual students with mild intellectual disabilities. The study has the following research questions:

Can a decoding intervention effectively improve word and nonword decoding for students with an intellectual disability?

Can a word decoding intervention effectively decrease the decoding errors of students with an intellectual disability?

## Methods

### Design

The current study used a single-subject design (SSD) to evaluate the effectiveness of an individualized decoding intervention for students with intellectual disabilities. Such a design involves collecting data on a single participant over time and comparing it to a baseline condition (Horner et al., 2005). Five baseline measurements were compared with five intervention measurements. SSD can be used to evaluate the effectiveness of an intervention for a particular individual (Horner et al., 2005). One advantage of using SSD is that it allows individualized interventions tailored to the specific needs and characteristics of the participant (Horner et al., 2005), which can be particularly beneficial for students with intellectual disabilities, as their reading abilities and needs may vary significantly (Katims, 2001). Using SSD, it is possible to identify whether an intervention is effective for individual students with intellectual disabilities and adjust the intervention for their individual progress.

### Settings

In Sweden, many students with intellectual disabilities attend compulsory school for pupils with learning disabilities. These schools often have teachers specially trained to teach students with intellectual disabilities. The students follow a curriculum for compulsory school for pupils with intellectual disabilities (Swedish National Agency for Education, 2011). In the first years, reading education should include strategies for decoding and comprehending words and student-oriented texts. The curriculum goals in the sixth grade are that the students should be able to read and understand various types of texts, such as factual texts, narratives, and poetry.

The current study was conducted in three compulsory schools for pupils with learning disabilities in Sweden. These three schools are comparable regarding the number of students (*n*=26–33), teachers (*n*=10-12), and recruitment area.

### Participants

In the current study, five students with mild intellectual disabilities in fourth grade participated. Three of them were boys, and two were girls. Their teachers selected them to benefit from individualized support to develop word decoding. According to the teachers, these students could decode words but needed support to acquire speed and accuracy in decoding. All participating students studied according to the curriculum for the compulsory school for pupils with intellectual disabilities (Swedish National Agency for Education, 2011). They were given the pseudonyms Axel, Ella, Fred, Iris, and Liam for this study. The students and their parents provided written informed consent to participate in the study.

### Measures

A standardized test measured the participants’ word and nonword decoding skills during baseline and intervention. The LäSt test (Elwér et al., 2016) consists of lists with words and nonwords. The words have increasing difficulty and length (from 2 to 8 letters). Both the word and nonword decoding tests include two parts. Each part should be read out loud and as quickly as possible, and the time for each part is 45 seconds. The number of correct read words in each part is summed. The word decoding test results can be between 0 and 200 correct words, whereas the number of correct decoded nonwords can be 0 to 100. In addition, the number of decoding errors was tallied for both word and nonword decoding. For students in Grade 4, the test-retest reliability is *r* = .88-.91 for word decoding and *r* = .74-.78 for nonword decoding. The mean score on the word decoding test is 119 (*SD*=27.5) and 65 (*SD*=20.0) on the nonword decoding test for students in Grade 4.

### Intervention

The Wolff Intensive Program (WIP) is an individual reading training that includes intensive phonics instruction combined with repeated reading and reading comprehension strategies (Wolff, 2015). The program is intended for students with reading and writing difficulties. It provides explicit decoding training to support students with reading disabilities in developing reading skills and is designed for one-to-one tutoring during an intensive and limited period. WIP has previously been evaluated on students in compulsory schools in Sweden (Wolff, 2016). According to the results, nine-year-olds with reading disabilities had gains in spelling, reading speed, reading comprehension, and phoneme awareness, which remained over a five-year follow-up.

The current study used two main components of the WIP program: (1) phonemic awareness and decoding training and (2) reading fluency training. The participating students were initially offered 12 sessions focusing on phonemic awareness and phonemic decoding training. After that, they received 13 sessions on reading fluency.

In the phonemic awareness and decoding training, the WIP includes teaching materials consisting of photos (consonants) and drawings (vowels) of mouths depicting the pronunciation of sounds. The mouth pictures were partly for awareness of articulation and partly for the benefit of phonemic decoding. For example, the students and teachers could ‘write’ words by putting strings of pictures of mouths onto the blackboard and asking each other to ‘read’ the mouths. Graphemes were matched with the mouths and sounded out into words. Gradually, the mouths were removed unless the student asked them to remain. Each session started with the student repeating the nine Swedish vowels, from the front vowels to the back vowels and from closed to open vowels. After the first week, this repetition took only around one minute. Here, the student works with minimal pairs, i.e., words with different semantic meanings and which only differ by one phoneme, e.g., log, dog.

The reading fluency sessions included repeated reading to strengthen the confidence and speed of the students' decoding skills. Each session ended with repeated reading of the same text. The number of words in the texts varied according to the student’s reading speed. The text was supposed to take the student four to five minutes to read the first time. During the first three weeks, one set of three texts with the same length and difficulty was used. The students recorded their progress by making graphs of the time spent reading the text and their accuracy. In the following three weeks, if appropriate, another set of three texts was chosen, and the student had improved reading speed with a larger number of words.

### Fidelity

The teachers followed a program with strict progression and detailed instructions for each session (Wolff, 2015). The sessions were provided by three special education teachers, who received instructions and training from the first author. All the intervention sessions were provided for each student individually and by one teacher, in addition to ordinary classroom teaching. Each session was planned for 30 minutes, and the participating students were supposed to receive three weekly sessions. After each intervention session, the teachers wrote a short daily report about the training and how long the session lasted. Weekly, the first author communicated with teachers and assessed the progress of the previous week's sessions. Any questions were addressed during this time. According to the logbook, all students received 25 intervention sessions, which lasted between 30-41 minutes (*M*=34, *SD*=3). Axel received 930 minutes of training, Ella received 905 minutes, Fred received 927 minutes, Iris received 940 minutes, and Liam received 916 minutes.

### Analyses

The data were analyzed with visual analysis and non-overlap of all pairs. The visual analysis refers to an inspection of the individual participant’s scores on decoding tests during the baseline and intervention sessions (Manolov et al., 2014). Such an analysis reveals whether each participant has a stable baseline and how the progress is related to the number of sessions in the intervention. Besides, the non-overlap of all pairs (NAP) shows data overlap between baseline and intervention phases (Parker & Vannest, 2009). The NAP is a probability score where 0.00-0.31 corresponds to weak effects, 0.32-0.84 to medium effects, and 0.85-1.00 to strong effects (Parker & Vannest, 2009). We used an online single-case effect size calculator for NAP calculations (Pustejovsky & Tipton, 2022).

## Results

**Figure 1.**
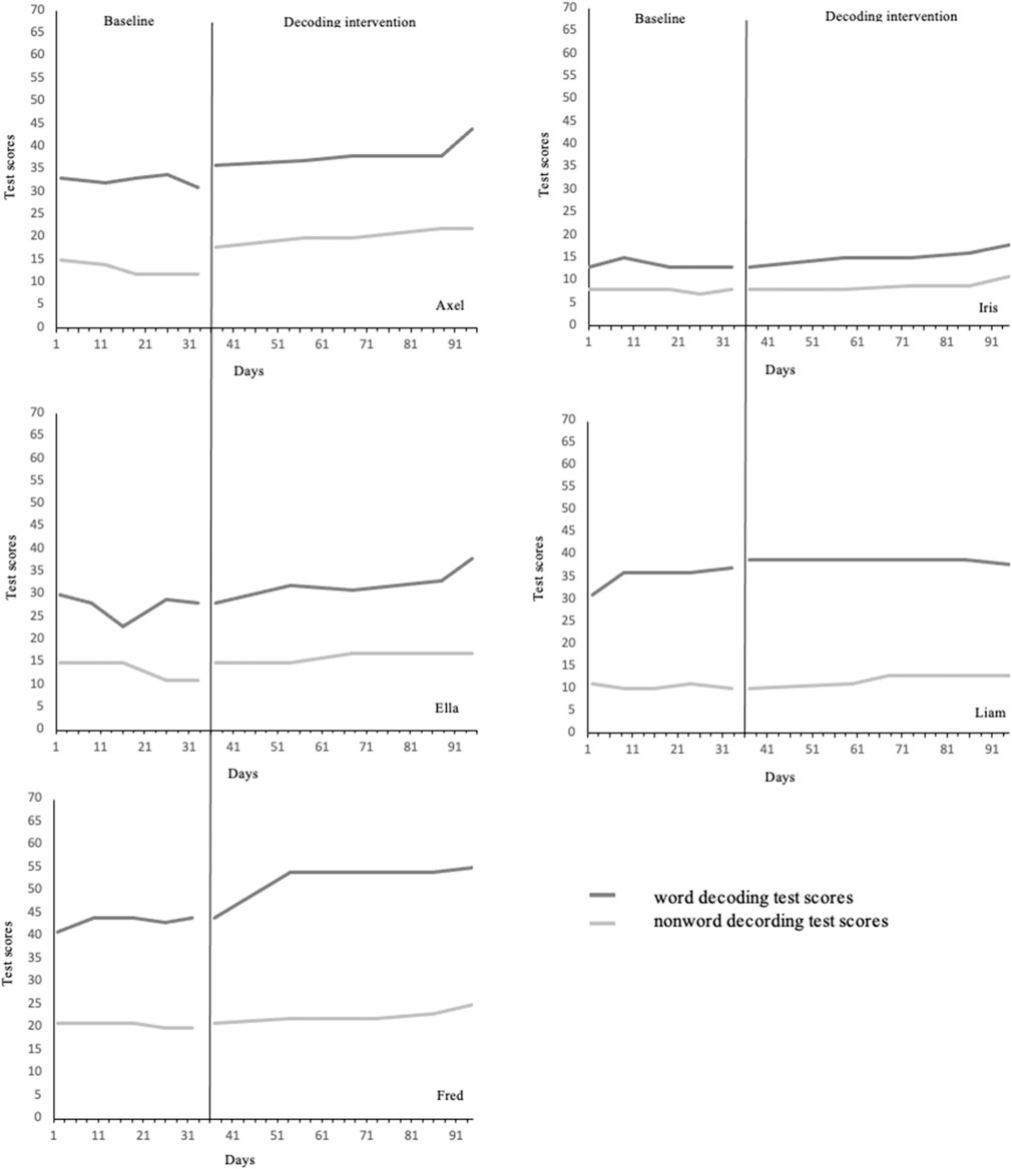
The five participants' scores on the word and nonword decoding test during baseline and intervention phases.

All participating students performed the five baseline and five intervention measurements. They also completed the 25 sessions within the word decoding intervention with the WIP. According to measurements in the baseline phase, the students demonstrated a small variability of the word and nonword decoding test scores (see Table 1). The small standard deviations reflect a stable baseline for each student. Similarly, the visual inspection of the data indicated a stable baseline for the five students (cf., Figure 1). Their performances on the word and nonword decoding tests improved during the intervention phase. However, there are different patterns among the students, and an initial positive trend was established among some of them (cf., Table 1).

**Table 1.** Participants’ scores and errors on decoding tests during baseline and intervention phases.

Dependent variables	Axel	Ella	Fred	Iris	Liam
	*M (SD)*	*M (SD)*	*M (SD)*	*M (SD)*	*M (SD)*
Word decoding					
Baseline scores	32.6 (1.1)	27.6 (2.7)	43.2 (1.3)	13.4 (0.9)	35.2 (2.4)
Intervention scores	38.6 (3.1)	32.4 (3.7)	52.2 (4.6)	15.4 (1.8)	38.8 (0.5)
Baseline errors	6.8 (0.5)	6.6 (0.9)	4.8 (0.5)	2.8 (0.5)	4.2 (0.8)
Intervention errors	2.8 (2.2)	4.2 (2.2)	2.0 (1.9)	2.2 (0.8)	2.8 (1.1)
Nonword decoding					
Baseline scores	13.0 (1.4)	13.4 (2.2)	20.6 (0.6)	7.8 (0.5)	10.4 (0.6)
Intervention scores	20.4 (1.7)	16.2 (1.1)	22.6 (1.5)	9.0 (1.2)	12.0 (1.4)
Baseline errors	3.6 (0.6)	5.6 (0.6)	3.0 (0.0)	3.4 (0.6)	3.0 (0.0)
Intervention errors	1.0 (0.0)	3.6 (1.1)	1.8 (0.8)	2.8 (0.5)	1.2 (0.5)

According to the measurements in the baseline phase, Axel had weak decoding skills. His performance on the word and nonword decoding tests corresponds to Stanine 1 for students in Grade 4. However, Axel’s test scores increased during the intervention phase, indicating that he decoded several words at a given time in the end (see Table 2). When comparing his highest scores in the baseline phase with the scores in the intervention phase, the average increase in word decoding was 14% and 36% in nonword decoding. A positive trend was established (see Table 2 and Figure 1). Besides, Axel gradually decreased his word decoding errors, and in the last measurement, he did not decode any words incorrectly.

**Table 2.** Participants’ percentage increase in test scores and decrease in decoding errors during the intervention phase.

Dependent variables	Axel	Ella	Fred	Iris	Liam
Word decoding score increase	**14%**	**8%**	**19%**	**3%**	**5%**
Measurement 1	6%	-7%	0%	-13%	5%
Measurement 2	9%	7%	23%	0%	5%
Measurement 3	12%	3%	23%	0%	5%
Measurement 4	12%	10%	23%	7%	5%
Measurement 5	29%	27%	25%	20%	3%
Word decoding errors decrease	**53%**	**30%**	**50%**	**-10%**	**7%**
Measurement 1	17%	0%	0%	-50%	-33%
Measurement 2	17%	0%	0%	-50%	-33%
Measurement 3	67%	17%	75%	0%	33%
Measurement 4	67%	50%	75%	0%	33%
Measurement 5	100%	83%	100%	50%	33%
Nonword decoding score increase	**36%**	**8%**	**8%**	**13%**	**9%**
Measurement 1	20%	0%	0%	0%	-9%
Measurement 2	33%	0%	5%	0%	0%
Measurement 3	33%	13%	5%	13%	18%
Measurement 4	47%	13%	10%	13%	18%
Measurement 5	47%	13%	19%	38%	18%
Nonword decoding errors decrease	**67%**	**28%**	**40%**	**7%**	**60%**
Measurement 1	67%	0%	0%	0%	33%
Measurement 2	67%	20%	33%	0%	67%
Measurement 3	67%	20%	33%	0%	67%
Measurement 4	67%	40%	67%	0%	67%
Measurement 5	67%	60%	67%	33%	67%

Ella showed weak word and nonword decoding skills in the baseline phase. Her word and nonword decoding test scores correspond to Stanine 1 for students in Grade 4. Ella’s decoding skills improved during the intervention. She decoded several words and nonwords with fewer errors. The average increase in the word and nonword decoding test was 8%. There was a considerable increase at the end of the intervention phase than at the beginning (cf., Table 2). Similarly, her decoding errors decreased more at the intervention’s end.

During the baseline phase, Fred was found to have challenges with word and nonword decoding. He is the participant with the highest scores on the decoding tests in the baseline phase. Thus, his performance corresponds to Stanine 1 for students in Grade 4. During the intervention phase, Fred improved both word and nonword decoding skills. His average increase in word decoding was 19%, and 8% in nonword decoding. The decrease in decoding errors was higher at the end of the intervention phase, as seen in Table 2.

Iris is the student with the weakest word and nonword decoding performance during the baseline phase (cf., Table 1). Her decoding skills correspond to Stanine 1 for students in Grade 4. She improved her decoding skills during the intervention phase. At the last measurement, her word decoding had increased by 20%, whereas the average increase was 3% on the word decoding test (cf., Table 2). Similarly, her nonword decoding increased by 38% at the last measurement, whereas the average increase was 13% on the nonword decoding test. In addition, she decoded words and nonwords with fewer errors at the end of the intervention phase.

During the baseline phase, Liam demonstrated weak word and nonword decoding. His decoding skills correspond to Stanine 1 for students in Grade 4. Like the other participants, he improved his decoding skills during the intervention phase. The average increase in word decoding was 5%, whereas the average increase in nonword decoding was 9%. Liam’s decoding errors decreased, especially in nonwords (see Table 2).

The analyses with NAP showed that the intervention with the WIP had medium to strong effects on the participating students' decoding skills (see Table 3). The intervention had a positive impact on each of the student's word and nonword decoding skills (NAP=0.72-1.00). They decoded several words at a given time and with fewer errors.

**Table 3.** Intervention effects on the students’ decoding skills.

	Axel			Ella			Fred			Iris			Liam		
	NAP	*SE*	95% CI	NAP	*SE*	95% CI	NAP	*SE*	95% CI	NAP	*SE*	95% CI	NAP	*SE*	95% CI
Test scores															
Word decoding	1.00	0.04	[1.00, 1.00]	0.88	0.13	[0.15, 0.98]	0.94	0.07	[0.57, 0.99]	0.84	0.14	[0.47, 0.97]	1.00	0.04	[1.00, 1.00]
Nonword decoding	1.00	0.04	[1.00, 1.00]	0.88	0.10	[0.50, 0.98]	0.94	0.07	[0.57, 0.99]	0.84	0.12	[0.47, 0.97]	0.82	0.15	[0.45, 0.96]
Decoding errors															
Word decoding	1.00	0.04	[1.00, 1.00]	0.88	0.10	[0.50, 0.98]	0.96	0.05	[0.59, 1.00]	0.72	0.17	[0.36, 0.92]	0.84	0.12	[0.47, 0.97]
Nonword decoding	1.00	0.04	[1.00, 1.00]	0.96	0.05	[0.59, 1.00]	0.90	0.11	[0.53, 0.98]	0.76	0.14	[0.39, 0.93]	1.00	0.04	[1.00, 1.00]

*Note*: NAP represents the non-overlap of all pairs. The NAPs for the test scores represent an increase in decoding, whereas the NAPs for the decoding errors represent a decrease in decoding errors for the participants.

## Discussion

The current study examined the impact of a word decoding intervention in Swedish on individual students with mild intellectual disabilities. During the baseline phase, the students demonstrated only a small variability in scores on the word and nonword decoding tests, i.e., the baseline showed stable results in decoding. After the baseline phase, all participating students received individualized instruction with the WIP during 25 sessions. The results showed that all five participating students improved their decoding of words in a given time, and their decoding errors decreased during the intervention phase. According to our findings a structured decoding intervention can enhance word decoding skills among students with mild intellectual disabilities.

However, our data showed different patterns among the students, indicating that they responded differently to the intervention. According to the effect sizes reported with NAP values, the students' results implied medium to strong effects on decoding words in a given time (NAP=0.84-1.00) and decreasing decoding errors (NAP=0.72-1.00). The increase reflected both decoding words and nonwords. Similarly, errors are decreased in the students’ word and nonword decoding during the intervention phase.

Moreover, our results revealed individual differences in the students’ progression in decoding. For example, Axel, Ella, and Iris showed weak decoding skills in the baseline phase (see Table 1) but made the most substantial progress during the intervention phase. Their improvement was more pronounced at the end of the intervention phase, as indicated by decreased decoding errors (cf., Table 2). Another pattern in progress was found in the results by Axel. His number of decoded words increased from the beginning of the intervention phase. He showed a positive trend in developing word and nonword decoding. Both Ella and Iris required more time to enhance the number of decoded words and nonwords in a given time than Axel. Besides, they also needed more sessions to decrease decoding errors than Axel. Subsequently, the students in the current study responded differently to the WIP intervention.

The use of intensive, systematic, and explicit instruction might explain the results of the current study. According to Wolff (2015), such instructions are beneficial for developing decoding in students. Previous research has also shown that explicit decoding interventions effectively improve reading skills for students with intellectual disabilities (Allor et al., 2014). Teachers using systematic and explicit instructions have an organized approach to teaching in which skills and concepts are taught logically and progressively. The teacher clearly explains and demonstrates the skills being taught. This type of instruction is reported to be effective in improving reading comprehension, particularly among struggling readers (e.g., Vellutino et al., 2000). Evidence suggests that decoding interventions can be effective among students with intellectual disabilities. For example, a meta-analysis on reading interventions demonstrated that phonics-based methods effectively improved decoding skills among persons with intellectual disabilities (Dessemontet et al., 2019). However, the effectiveness of decoding interventions may vary depending on the individual student's specific needs and abilities. Students with intellectual disabilities may also need more intensive and explicit instruction in decoding to acquire reading skills (Alnahdi, 2015).

Besides using intensive, systematic, and explicit instructions, the content of the applied intervention with the Wolff Intensive Program (WIP, Wolff, 2015) might explain the positive results. The WIP focuses on phonemic awareness and phonics. By providing phonics instruction to the participating students, they were given opportunities to develop phonemic awareness essential for learning to decode words (Johnston et al., 2012; National Reading Panel, National Institute of Child Health & Human Development (US), 2000). According to Wolff (2015), the content of the WIP, combined with intensive, systematic, and explicit instruction, might support students with intellectual disabilities to develop the skills they need to advance to the consolidated alphabetic stage (cf., Frith's model from 1985). The WIP is, to our knowledge, not previously evaluated on students with intellectual disabilities, but our findings support Wolff's assumption that the WIP might benefit students with mild intellectual disabilities. Teaching the participating students to decode words using phonemic awareness and phonics included in the WIP, the interventions supported them in decoding more words accurately and with fewer errors.

Furthermore, the results of the current study can be discussed in relation to the theoretical model of decoding development proposed by Frith (1985). According to this model, students go through several stages as they develop their reading skills. In the alphabetic stage, they begin to understand that words consist of different sounds that can be combined to create different words. This phonemic awareness is a critical foundation for decoding. When students understand phoneme-grapheme correspondences well, they should be able to decode words by sounding them out based on their knowledge of the letter-sound relationships. Therefore, it is likely that the students in the current study who are more secure in phoneme-grapheme correspondences also showed improved decoding skills.

Since students with intellectual disabilities are a very heterogeneous group, an SSD was used in this study; rather than making group comparisons, we closely followed five students. Generally, SSD is a valuable method for evaluating the effectiveness of individualized reading interventions for students with intellectual disabilities. It is a method that can help identify strategies most effective for improving reading skills among students with disabilities. Overall, the results of this study suggest that the WIP intervention might be effective in enhancing decoding skills among students with mild intellectual disabilities, but the impact varies.

### Limitations

Although the current study showed that intervention with the WIP could positively affect decoding skills in students with mild intellectual disabilities, there are some limitations to consider. For example, the intervention was delivered by different teachers, which could have affected the results as there might be a teacher effect rather than an intervention effect (cf., Dobber et al., 2017). However, to increase the study's internal validity, the teachers were all educated by the first author on the WIP. The teachers followed the same manual and filled in a logbook after each session. In the logbook, they wrote which parts of the WIP the student worked on, how long the session took, and how it went. During the intervention, the first author contacted the teachers every week. According to the notes in the logbook, the five students received about 34 minutes of intervention during each session, and all five completed the 25 sessions. Since this is, to our best knowledge, the first study focusing on enhancing decoding in Swedish in young primary school students with intellectual disabilities, the WIP needs to be further investigated.

### Practical implications

Evidence suggests that students with intellectual disabilities may require more intervention time and explicit instruction in decoding to develop reading skills in languages other than English. This structure of different orthographies varies, and students with intellectual disabilities may need additional support to learn the specific phonemic and phonological rules of the language they are learning to read (Fuchs et al., 2013). Studies conducted in English have found that students with intellectual disabilities benefit from systematic and explicit instruction in phonemic awareness and phonics (Allor et al., 2014). Such instructions involve understanding the individual sounds that make words and the relationships between those sounds and written letters. According to results from the current study, teaching decoding skills to students with mild intellectual disabilities should involve specific phonemic and phonological rules in Swedish and using multisensory and visual supports to facilitate learning.

Although a word-decoding intervention, such as the WIP, could demonstrate a strong effect on students’ decoding skills, their actual skills may have improved marginally. Therefore, teachers should evaluate the clinical significance of the intervention on the individual student’s decoding skills and determine how long it is reasonable to continue with such an intervention. The teacher should consider whether the student with mild intellectual disabilities benefits from supplemental decoding interventions to develop alphabetic and autonomous decoding (cf., Frith, 1985) or from other types of efforts to be able to assimilate text (cf., Afacan et al., 2018; Perelmutter et al., 2017; Svensson et al., 2019). For instance, assistive technology such as text-to-speech might support students in assimilating text through listening. Students with mild intellectual disabilities might benefit from developing their decoding and using text-to-speech to have better prerequisites to assimilate meaningful and more complex texts. Thus, more research is needed regarding the effects of using the WIP and assistive technology for students with mild intellectual disabilities.
